# Real-world experience with sodium-glucose cotransporter 2 inhibitors in adults with Fontan circulatory failure

**DOI:** 10.3389/fcvm.2026.1771868

**Published:** 2026-05-08

**Authors:** Ralph M. L. Neijenhuis, Ari M. Cedars, Ali Zaidi, Guillermo Torres-Viera, Renata Mazurek, Niki L. Walker, Filip Zemrak, Simon T. MacDonald, Amanda Hunter, Philippine Kiès, Lena Bosch, Lorna Swan, Heleen B. van der Zwaan, J. Wouter Jukema, Monique R. M. Jongbloed, Gruschen R. Veldtman, Anastasia D. Egorova

**Affiliations:** 1 Department of Cardiology, Leiden University Medical Center, Leiden, Netherlands; 2 CAHAL, Center for Congenital Heart Disease Amsterdam-Leiden, Leiden University Medical Center, Leiden, Netherlands; 3 Congenital Heart Disease Network (NAH), Leiden University Medical Center, Leiden, Netherlands; 4 Divisions of Pediatric and Adult Cardiology, Johns Hopkins University, Baltimore, MD, United States; 5 Mount Sinai Adult Congenital Heart Disease Center, Mount Sinai Fuster Heart Hospital, New York, NY, United States; 6 Scottish Adult Congenital Cardiac Service (SACCS), Golden Jubilee University National Hospital, Glasgow, United Kingdom; 7 Barts Heart Centre, St Bartholomew’s Hospital, Barts Health NHS Trust, London, United Kingdom; 8 Institute of Cardiovascular Science, University College London, London, United Kingdom; 9 Department of Cardiology, University Hospital of Wales, Cardiff, United Kingdom; 10 Department of Cardiology, University Medical Center Utrecht, Utrecht, Netherlands; 11 Congenital Heart Disease Network (NAH), University Medical Center Utrecht, Utrecht, Netherlands; 12 Netherlands Heart Institute, Utrecht, Netherlands; 13 Department of Anatomy & Embryology, Leiden University Medical Center, Leiden, Netherlands; 14 Adult Congenital Heart Disease Center, Helen DeVos Children’s Hospital, Grand Rapids, MI, United States

**Keywords:** adult congenital heart disease (ACHD), FCF, heart failure, SGLT2I, single ventricle, univentricular

## Abstract

**Background:**

Patients with Fontan physiology frequently develop Fontan circulatory failure (FCF), but there are no evidence-based pharmacological treatment options.

**Objectives:**

This study evaluated the safety and efficacy of sodium-glucose cotransporter 2 inhibitors (SGLT2i) in FCF.

**Methods:**

A real-world, multicenter study of all adult FCF patients included in the international ACHIEVE-SGLT2i registry (NCT06932081) was conducted. Data on side effects, treatment discontinuation, and clinical outcomes were collected. Longitudinal changes in serum biomarkers and clinical parameters from one year before to one year after SGLT2i initiation were evaluated using linear mixed models. Responses between patients with reduced (FCFrEF) vs. preserved ventricular function (FCFpEF) were compared.

**Results:**

Thirty-three FCF patients were started on SGLT2i between January 2017 and October 2024. The median age was 32 [20.5–42] years, 17 (51.5%) were female, 11 (33.3%) had FCFrEF, and 22 (66.7%) FCFpEF. Over a median follow-up of 8.0 [3.2–12.2] months, 5 (15.2%) patients reported side effects, of whom 3 (9.1%) permanently discontinued SGLT2i. There were 11 FCF-related hospitalizations in the year before SGLT2i and 7 during follow-up in 9 patients. Blood pressure and renal function remained stable. NT-proBNP increased from 186.3 [116.8–297.1] to 272.6 [180.7–411.3] ng/L in the year before treatment (+46.4%, *p* = 0.022). In the year after starting SGLT2i, NT-proBNP levels decreased significantly to 200.4 [126.5–317.4] ng/L (−26.5%, *p* = 0.010), in both FCFrEF and FCFpEF patients.

**Conclusions:**

SGLT2i were safe and well-tolerated in adult patients with FCF. SGLT2i treatment was associated with a reduction in NT-proBNP, regardless of FCF phenotype.

**Clinical Trial Registration:**

ClinicalTrials.gov, identifier NCT06932081.

## Graphical Abstract

**Figure:**
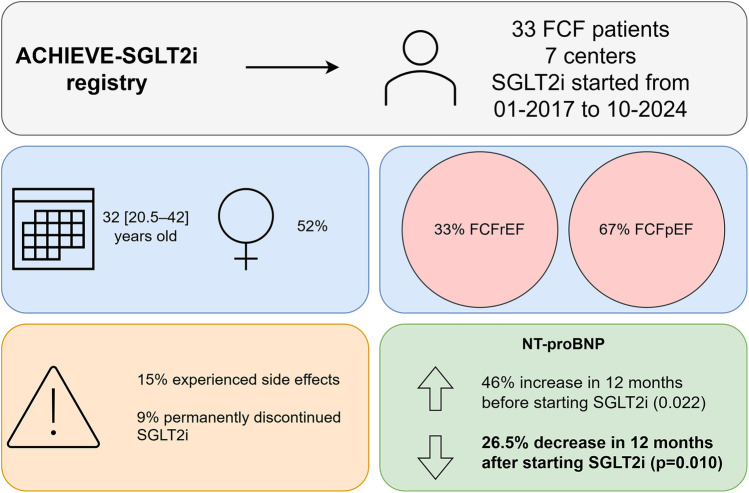
SGLT2i in adults with fontan circulatory failure. In this real-world multicenter evaluation of the use of SGLT2i in 33 adult FCF patients included in the ACHIEVE-SGLT2i registry, SGLT2i were safe and well-tolerated. SGLT2i treatment was associated with NT-proBNP reduction, regardless of FCF phenotype. FCF, Fontan circulatory failure; FCFpEF, Fontan circulatory failure with preserved ejection fraction; FCFrEF, Fontan circulatory failure with reduced ejection fraction; NT-proBNP, N-terminal pro-B-type natriuretic peptide; SGLT2i, sodium-glucose cotransporter 2 inhibitors.

## Introduction

The Fontan operation, first performed in 1968 for patients with tricuspid atresia, has given patients with a single functional ventricle a viable chance at long-term survival without early transplant (1). This monumental achievement in the management of patients with severe congenital heart disease has resulted in a rapidly expanding group of patients living with a Fontan circulation. In 2020, the estimated prevalence was 66 per million, with the majority (55%) being adults (2). This growing group of adult Fontan patients suffers the sequelae of a circulation predicated on chronically elevated systemic venous pressure and reduced cardiac output. Long-term exposure to these physiologic stressors results in the majority of Fontan patients experiencing difficulties in carrying out their daily life activities. Fontan circulatory failure (FCF) is the umbrella term used to describe this state, characterized by a heterogeneous underlying etiology that frequently includes systolic and diastolic ventricular dysfunction, atrioventricular valve failure, and increased pulmonary vascular resistance (3). The estimated overall prevalence of FCF is 30%, and approaches 100% in patients over 50 years of age (4).

Due to the heterogeneous pathophysiological mechanisms underlying FCF, extrapolation of the guidelines for the management of conventional acquired heart failure is inappropriate (5). Consequently, the identification of medical therapies that could benefit FCF patients has been identified as one of the key high-impact research questions in both the American and European adult congenital heart disease (ACHD) guidelines (5, 6). Sodium-glucose cotransporter 2 inhibitors (SGLT2i) have become one of the four established “pillars” in the treatment of conventional heart failure, and the pleiotropic mechanisms by which SGLT2i are hypothesized to exert their beneficial effects makes them an interesting pharmacological treatment option for FCF (7). In addition to their direct glucosuric and natriuretic properties, a range of off-target effects have been proposed, including improvements in cardiac mitochondrial function, reductions in cardiac fibrosis and oxidative stress, and enhancement of endothelial function (7–9). The latter is of particular interest, given the central role of endothelial dysfunction in FCF (10).

A few small studies have evaluated the use of SGLT2i in the Fontan population and reported promising results (11–16). These studies may, however, be subject to publication bias, leaving the need for real-world experience and larger sample sizes to assess the clinical efficacy. In addition, the limited data available has shown that SGLT2i therapy may improve systolic ventricular function in single ventricle patients, and there may be a difference between patients with reduced and preserved ventricular function (15). In view of these data, the present study aims to evaluate the safety and efficacy of SGLT2i in adults with FCF included in the international, real-world ACHIEVE-SGLT2i registry, and investigates differential responses between FCF patients with a reduced ejection fraction (FCFrEF) and preserved ejection fraction (FCFpEF).

## Materials and methods

### Study design

In this retrospective cohort study, all adults (≥18 years old) with FCF started on an SGLT2i and included in the international, real-world **A**dult **C**ongenital **H**eart disease **I**nternational **EV**aluation of the **E**ffectiveness of **SGLT2i** (ACHIEVE-SGLT2i) registry (NCT06932081), were eligible for inclusion.

The diagnosis of FCF was made by the treating cardiologist, based on a previously established extrapolation of the universal definition of heart failure to the Fontan circulation (3, 17). Patients were classified as either FCFrEF (≥moderately reduced systolic ventricular function) or FCFpEF (≤mildly reduced systolic ventricular function) at SGLT2i initiation. This dichotomization was based on qualitative assessment of ventricular function at baseline transthoracic echocardiography, consisting of a combination of strain measurement, fractional area change, ejection fraction, and visual assessment. Data were collected retrospectively from the electronic health records at the participating centers, from 1 year before initiation of SGLT2i therapy onwards, and at each subsequent visit and/or hospitalization, as previously specified (18).

Appropriate medical ethical board approval was obtained at all participating centers [coordinating center: Leiden University Medical Center Medical Research Involving Human Subjects Act (WMO) committee division 1 protocol reference 2022-068]. The study was conducted in accordance with the principles of the 2013 Declaration of Helsinki, and complied with the STROBE (STrengthening the Reporting of OBservational studies in Epidemiology) statement (19).

### Outcomes

Safety and tolerability of SGLT2i therapy were evaluated by assessing side effects, (reason for) treatment discontinuation, changes in systolic and diastolic blood pressure (mmHg) and changes in renal function measured with serum creatinine (µmol/L).

The primary efficacy outcome metric was change in the heart failure surrogate biomarker N-terminal pro–B-type natriuretic peptide (NT-proBNP, ng/L). Secondary efficacy outcomes were changes in: hemoglobin (mmol/L), hematocrit (L/L), albumin (g/L), aspartate transaminase (AST, U/L), alanine transaminase (ALT, U/L), alkaline phosphatase (ALP, U/L), gamma-glutamyltransferase (GGT, U/L), and body weight (kg). Differences in these outcomes between FCFrEF and FCFpEF patients were assessed.

Data on FCF-related hospitalizations and mortality were also collected. An FCF-related hospitalization was defined as a hospital admission crossing a calendar day, with at least one of the following: 1) management of new or worsening heart failure symptoms with the initiation or increase of pharmacotherapy for FCF (as specified below), or intravenous inotropes, 2) management of ascites (including drainage), 3) management of lymphatic dysfunction.

Concomitant longitudinal changes in the following classes of pharmacotherapy for FCF were assessed, as potential confounders for evaluating the efficacy of SGLT2i therapy: 1) angiotensin-converting enzyme inhibitors, angiotensin receptor-neprilysin inhibitors, or angiotensin receptor blockers, 2) mineralocorticoid receptor antagonists, 3) beta blockers, 4) diuretics, and 5) pulmonary vasodilators including phosphodiesterase type 5 inhibitors, prostacyclin analogs, and endothelin-1 receptor antagonists.

### Statistical analysis

All statistical analyses were performed with SPSS v25 (IBM Corp, Armonk, NY, USA) and R Statistical Software (v4.4.2; R Core Team 2023). Normally distributed continuous variables were presented as mean ± standard deviation and compared with unpaired *t*-tests. Non-normally distributed continuous variables were presented as median [Q1–Q3] and compared with Mann–Whitney *U* tests. Binary and categorical variables were presented as frequencies with percentages in parentheses, and compared with Chi-square, Fisher's exact tests, or McNemar's tests, as appropriate.

Linear mixed models were constructed using the “nlme” package (v3.1.167; R Core Team 2024) to account for unbalanced repeated measurements data, as both the number of observations per participant and the timing of follow-up assessments varied across individuals. To assess if the trajectory of an outcome variable changed after initiation of SGLT2i, time from day −365 to day +365 was included as a linear continuous variable, and an interaction term with a binary variable for treatment (on SGLT2i yes or no) was included as a fixed effect. To assess the potential difference in response to SGLT2i between FCFrEF and FCFpEF patients, the contribution of FCF phenotype as a significant binary covariate was tested. Due to the expected confounding effect of protein-losing enteropathy (PLE) on serum albumin, PLE status was included as a binary covariate in the model evaluating albumin changes. Random intercept models, random intercepts and slopes models, and different variance and correlation structures were tested to select the best-fitting model. Model selection was based primarily on the Bayesian information criterion (BIC), with secondary consideration of the Akaike information criterion (AIC) or likelihood ratio tests when appropriate. Model parsimony was prioritized over predictive capacity and fit was verified through residual analysis. If the normalized residuals of the model showed significant deviation from normality, a Log_10_ transformation of the outcome variable was performed and normality was re-assessed. The fixed effects were visualized with corresponding 95% confidence intervals (CIs) for fixed-effect variance. To enhance clinical interpretability, the results were expressed as estimated values at day −365, day 0, and day 365, with 95% CIs. The percentages change in the year before and after starting SGLT2i were calculated. A two-sided *p*-value ≤0.05 was considered statistically significant.

## Results

In total, 33 FCF patients who started an SGLT2i between January 2017 and October 2024 were identified at 7 tertiary ACHD centers in the Netherlands, United Kingdom, and United States, and included in the study (Supplementary Figure 1). The median age was 32 [20.5–42] years, 17 (51.5%) were female, and 1 (3%) had type 2 diabetes mellitus. Eleven patients (33.3%) were categorized as FCFrEF, and 22 (66.7%) as FCFpEF. There were no significant differences in the baseline characteristics between FCF phenotypes (Table 1).

**Table 1:** Baseline characteristics.

Characteristics	All (*n* = 33)	FCFrEF (*n* = 11)	FCFpEF (*n* = 22)	*P*-value
Age, y	32 [20.5–42]	21 [20–41]	34 [22.5–43.5]	0.329
Sex, female	17 (51.5)	5 (45.5)	12 (54.5)	0.721
*Type and dose of SGLT2i*				0.712
Dapagliflozin	18 (54.5)	7 (63.6)	11 (50)	
10 mg OD	16 (48.5)	7 (63.6)	9 (40.9)	
5 mg OD	2 (6.1)	0	2 (9.1)	
Empagliflozin 10 mg OD	15 (45.5)	4 (36.4)	11 (50)	
*Anatomy*				0.245
RV dominant (hypoplastic left heart syndrome)	10 (30.3)	5 (45.5)	5 (22.7)	
RV dominant (other)	5 (15.2)	2 (18.2)	3 (13.6)	
LV dominant	15 (45.5)	3 (27.3)	12 (54.5)	
Biventricular	2 (6.1)	0	2 (9.1)	
Mixed/indeterminate ventricle	1 (3)	1 (9.1)	0	
*Fontan type*				0.225
Extracardiac Fontan	16 (48.5)	7 (63.6)	9 (40.9)	
Lateral tunnel	10 (30.3)	4 (36.4)	6 (27.3)	
Atriopulmonary Fontan	6 (18.2)	0	6 (27.3)	
Other (bidirectional cavopulmonary connection + interrupted left-sided IVC + hepatic veins connected directly to PA)	1 (3)	0	1 (4.5)	
FCF-related hospitalization in the year preceding SGLT2i	8 (24.2)	2 (18.2)	6 (27.3)	0.687
SGLT2i started during FCF-related hospitalization	4 (12.1)	1 (9.1)	4 (18.2)	0.643
Protein-losing enteropathy	2 (6.1)	0	2 (9.1)	0.542
Type 2 diabetes mellitus (on oral therapy)	1 (3)	0	1 (4.5)	1.000
CIED	9 (27.3)	2 (18.2)	7 (31.8)	0.681
*CIED type*				1.000
DDD pacemaker	5 (15.2)	1 (9.1)	4 (18.2)	
AAI pacemaker	3 (9.1)	1 (9.1)	2 (9.1)	
S-ICD	1 (3)	0	1 (4.5)	
Physical examination findings				
Weight, kg (*n* = 31)	72.4 ± 12.5	72 ± 12.2	74 ± 12.4	0.674
BMI, kg/m^2^ (*n* = 31)	26.5 ± 4.6	25.3 ± 4.4	27.6 ± 4.7	0.220
Heart rate, bpm (*n* = 31)	73 ± 11	76.6 ± 11.4	71.1 ± 10.8	0.188
Oxygen saturation, % (*n* = 30)	94 [91.8–96]	94.5 [92–96.3]	94 [91.3–95.8]	0.491
Systolic blood pressure, mmHg (*n* = 31)	111 ± 12	113 ± 10	110 ± 12	0.519
Diastolic blood pressure, mmHg (*n* = 31)	66 [61–74]	69 [66–76]	64 [60–73]	0.280
Laboratory parameters				
Hemoglobin (mmol/L)	9.7 [8.7–10.2]	10 [9.4–10.9]	9.6 [8.1–10]	0.058
Hematocrit (L/L)	0.452 ± 0.045	0.475 ± 0.038	0.440 ± 0.045	0.078
Mean corpuscular volume (fL)	88.7 [87.2–92.6]	88.6 [87.7–91.8]	89.7 [83.3–92.8]	0.927
Creatinine (μmol/L) (*n* = 29)	72 ± 12	75 ± 14	71 ± 11	0.389
eGFR, mL/min/1.73m^2^ (*n* = 27)	73 [60–98]	60 [60–90]	84[60–105]	0.147
eGFR ≥60 mL/min/1.73m^2^ (*n* = 27)	27 (100)	9 (100)	18 (100)	—
NTproBNP (ng/L) (*n* = 17)	240.2 [160.6–482.6]	255.3 [164.2–487.4]	220.8 [111.5–534.5]	0.773

Values are *n* (%), mean ± standard deviation, or median [Q1–Q3]. Independent non-parametric data were compared with the Mann–Whitney *U* test and parametric data with the unpaired *t*-test. Independent categorical data were compared with the Chi-square or Fisher's exact test, as appropriate. Data were available for all patients unless specified otherwise. BMI, body mass index; CIED, cardiac implantable electronic device; eGFR, estimated glomerular filtration rate; FCF, Fontan circulatory failure; FCFpEF, Fontan circulatory failure with preserved ejection fraction; FCFrEF, Fontan circulatory failure with reduced ejection fraction; HF, heart failure; IVC, inferior vena cava; LV, left ventricle; OD, once daily; PA, pulmonary artery; RV, right ventricle; S-ICD, subcutaneous implantable cardioverter defibrillator; SGLT2i, sodium-glucose cotransporter 2 inhibitor.

### Safety and tolerability

**Figure 1:**
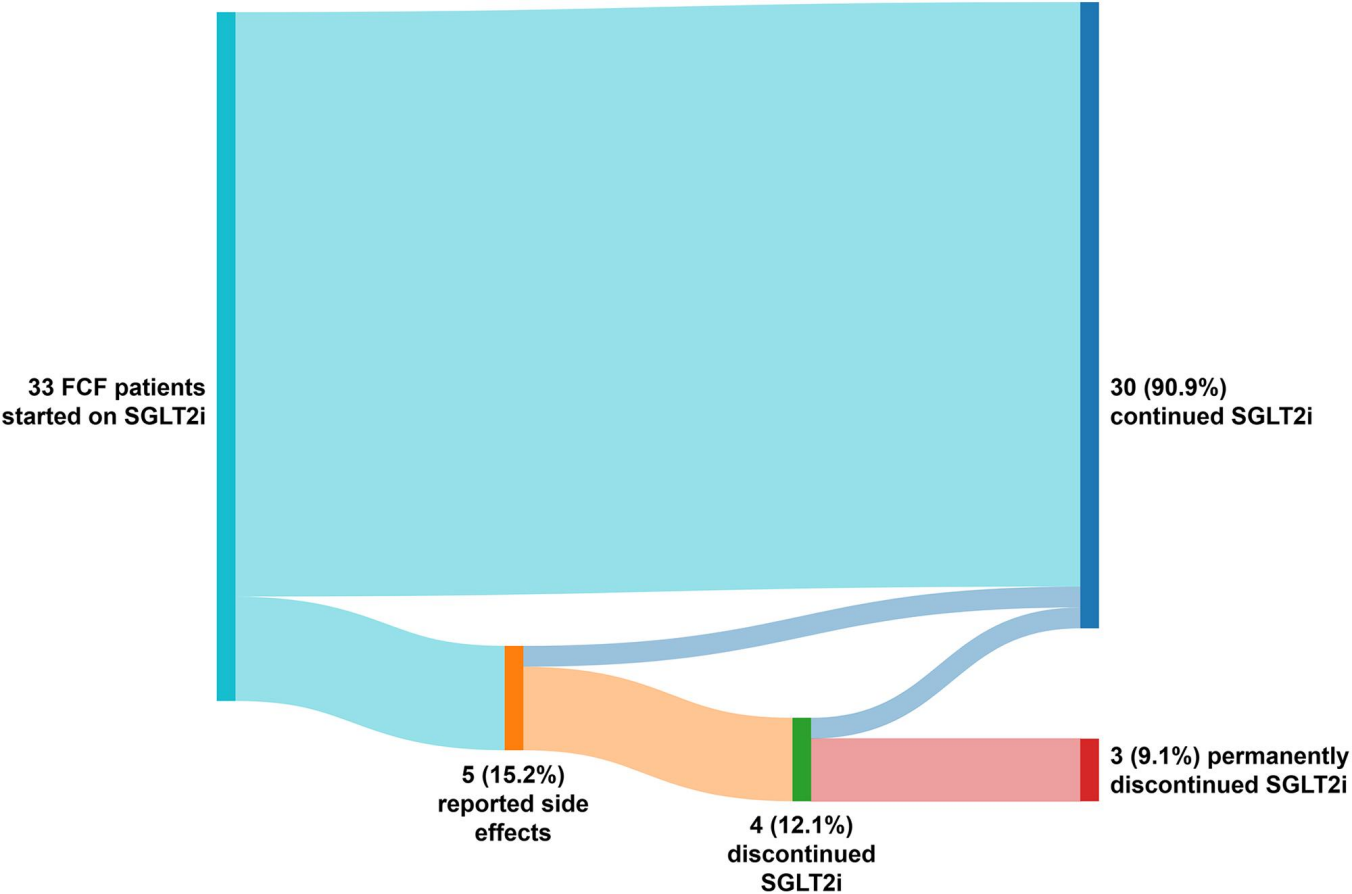
Safety and tolerability of SGLT2i. Side effects and discontinuation of SGLT2i therapy in 33 FCF patients over a median follow-up of 8.0 [3.2–12.2] months. FCF, Fontan circulatory failure; SGLT2i, sodium-glucose cotransporter 2 inhibitor.

The majority were started on dapagliflozin (*n* = 18, 54.5%). The recommended dose of 10 mg once daily was prescribed in most patients (*n* = 31, 93.9%). SGLT2i was initiated during an FCF-related hospitalization in 4 (12.1%) patients. Over a median follow-up duration of 8.0 [3.2–12.2] months, 5 patients (15.2%) experienced side effects, of whom 3 (9.1%) permanently discontinued SGLT2i therapy (Figure 1). The reported side effects are presented in Supplementary Table 1. One patient switched from dapagliflozin to empagliflozin due to abdominal discomfort, after which the complaints resolved. A patient with type 2 diabetes mellitus switched from dapagliflozin 10 mg once daily to empagliflozin 25 mg once daily after 1 year due to persistently elevated HbA1c values. No other SGLT2i therapy changes occurred.

At baseline, the mean systolic blood pressure was 111 ± 12 mmHg and the median diastolic blood pressure was 66 [61–74] mmHg. No significant changes after starting SGLT2i were observed (*p* = 0.443 and *p* = 0.680 respectively), nor were there differences between FCFrEF and FCFpEF. All patients had a creatinine-based estimated glomerular filtration rate (eGFR) of at least 60 mL/min/1.73m^2^ at baseline. Serum creatinine did not change significantly after starting SGLT2i (*p* = 0.648) and there were no differences between FCF phenotypes. The estimates for all mixed models are presented in Table 2. The mixed model outputs are presented in Supplementary Table 2 and the characteristics in Supplementary Table 3.

**Table 2:** Mixed models yearly estimates and changes.

Parameters	Estimate	95% CI	% change^a^	*P*-value
Safety				
Creatinine (µmol/L)				
Day −365	68.340	62.127–74.552	+7.2	0.082
Day 0	73.272	68.262–78.282		
Day 365	80.715	72.264–89.166	+10.1	0.648
Systolic blood pressure (mmHg)				
Day −365	117.904	109.693–126.110	−6.6	0.101
Day 0	110.136	106.001–114.271		
Day 365	107.744	100.666–114.821	−2.2	0.443
Diastolic blood pressure (mmHg)				
Day −365	67.242	61.184–73.301	−0.7	0.894
Day 0	66.783	63.699–69.867		
Day 365	64.199	58.966–69.433	−3.9	0.680
Efficacy				
NT-proBNP (ng/L)^b^				
Day −365	186.255	116.779–297.064	46.4	**0** **.** **022**
Day 0	272.648	180.718–411.342		
Day 365	200.391	126.524–317.381	−26.5	**0** **.** **010**
Hemoglobin (mmol/L)				
FCFrEF				
Day −365	9.814	9.061–10.566	+3.1	0.224
Day 0	10.115	9.413–10.818		
Day 365	10.526	9.763–11.288	+4.1	0.793
FCFpEF				
Day −365	8.792	8.173–9.411	+3.4	0.224
Day 0	9.093	8.565–9.622		**0** **.** **025**
Day 365	9.504	8.873–10.134	+4.5	0.793
Hematocrit (L/L)				
FCFrEF				
Day −365	0.466	0.435–0.496	+3.7	0.106
Day 0	0.483	0.455–0.511		
Day 365	0.508	0.477–0.539	+5.2	0.656
FCFpEF				
Day −365	0.425	0.400–0.451	+4.1	0.106
Day 0	0.442	0.421–0.464		**0** **.** **025**
Day 365	0.468	0.442–0.493	+5.7	0.656
Albumin (g/L)				
No PLE				
Day −365	44.082	41.400–46.765	+5.4	0.097
Day 0	46.447	44.389–48.506		
Day 365	46.436	43.659–49.213	<−0.1	0.318
PLE				
Day −365	34.444	27.653–41.235	+6.9	0.097
Day 0	36.809	30.252–43.366		**0** **.** **009**
Day 365	36.798	30.157–43.439	<−0.1	0.318
AST (U/L)				
Day −365	24.204	20.006–28.402	+8.2	0.322
Day 0	26.191	22.971–29.411		
Day 365	26.080	21.883–30.277	−0.4	0.523
ALT (U/L)				
FCFrEF				
Day −365	28.821	23.178–34.465	−2.6	0.710
Day 0	28.083	22.975–33.190		
Day 365	31.718	25.916–37.520	+12.9	0.184
FCFpEF				
Day −365	21.401	16.877–25.925	−3.4	0.710
Day 0	20.663	17.075–24.251		**0** **.** **022**
Day 365	24.298	19.707–28.888	+17.6	0.184
ALP (U/L)^b^				
Day −365	90.713	77.683–105.930	−0.6	0.921
Day 0	90.152	78.888–103.024		
Day 365	100.916	86.270–118.048	+11.9	0.255
GGT (U/L)^b^				
Day −365	56.753	41.740–77.164	+15.3	0.277
Day 0	65.427	52.067–82.215		
Day 365	87.848	67.627–114.117	+34.3	0.426
Weight (kg)				
Day −365	71.714	66.854–76.574	+2.4	0.379
Day 0	73.426	69.443–77.409		
Day 365	75.142	70.735–79.549	+2.3	0.999

ALP, alkaline phosphatase; ALT, alanine transaminase; AST, aspartate transaminase; FCF, Fontan circulatory failure; FCFpEF, Fontan circulatory failure with preserved ejection fraction; FCFrEF, Fontan circulatory failure with reduced ejection fraction; GGT, gamma-glutamyltransferase; NT-proBNP, N-terminal pro–B-type natriuretic peptide.

a The percentage change after the “Day −365” estimate is calculated as the percentage change from the day 0 estimate to the day −365 estimate: (“day 0”–“day −365”)/“day −365” * 100). The change after the ‘Day 365 estimate is calculated in a similar fashion.

b These models were fitted on a log10 transformed variable due to significant departure from normality of the normalized residuals for the normal scale variable. In this table, the estimates have been back-transformed to the original scale for interpretation.

### Efficacy

**Figure 2:**
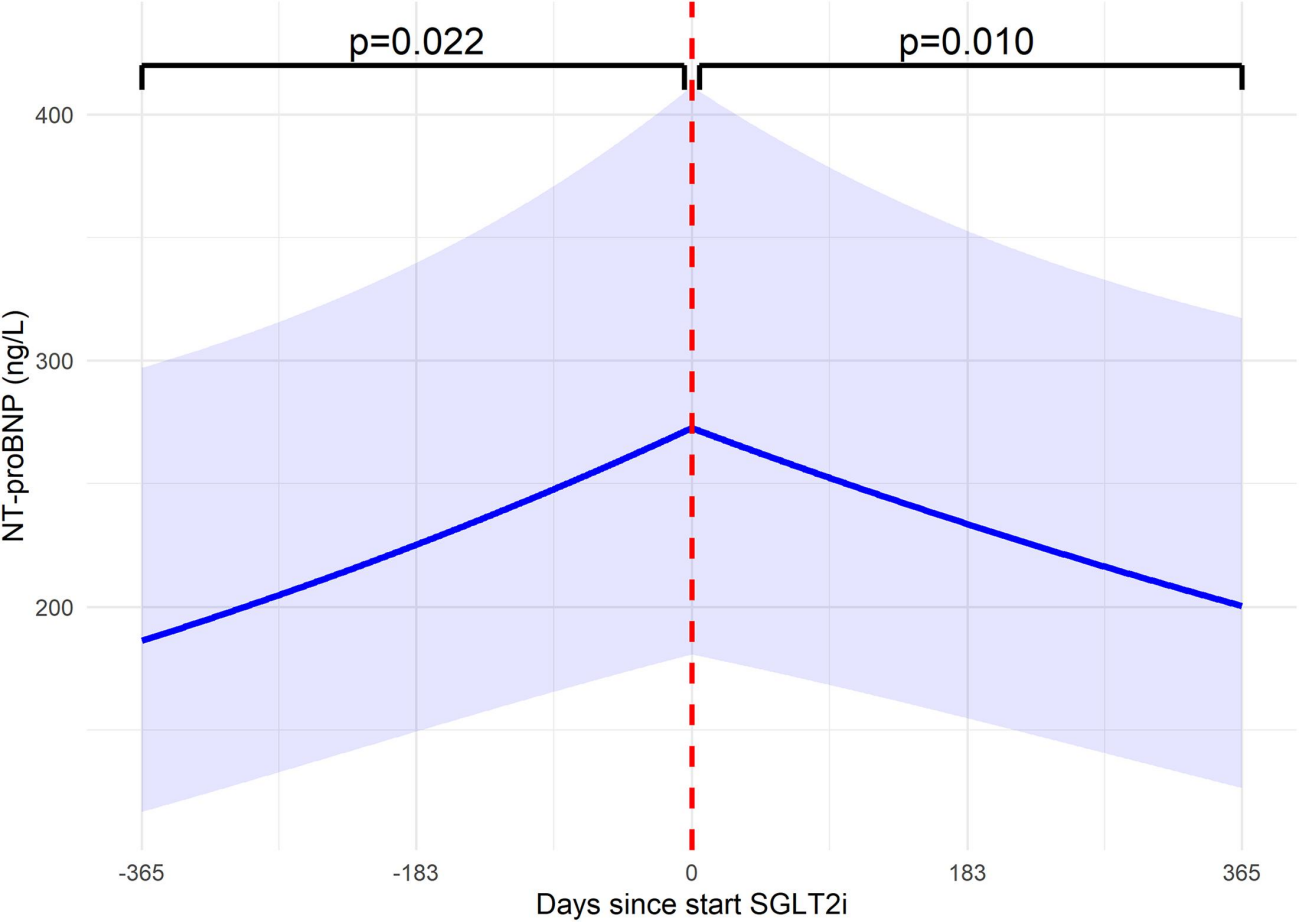
NT-proBNP changes in the year before and after starting SGLT2i. Changes in NT-proBNP (ng/L) in the year before and after starting SGLT2i. In the year before starting SGLT2i, there was an estimated 46.4% increase in NT-proBNP (*p* = 0.022). In the year after starting, there was a significant reversal of the trajectory, with an estimated decrease of 26.5% (*p* = 0.010). This indicates that starting SGLT2i significantly impacted and reversed NT-proBNP. The dashed red line indicates when SGLT2i therapy was started. NT-proBNP, N-terminal pro–B-type natriuretic peptide; SGLT2i, sodium-glucose cotransporter 2 inhibitor.

In the year before starting SGLT2i, NT-proBNP increased by 46.4%, from 186.3 [116.8–297.1] to 272.6 [180.7–411.3] ng/L (*p* = 0.022). In the year after starting SGLT2i, this trend reversed, and a significant 26.5% decrease in NT-proBNP to 200.4 [126.5–317.4] ng/L (*p* = 0.010) was observed (Figure 2). There were no significant differences in this response between FCFrEF and FCFpEF patients.

There were no significant changes in the year before or after starting SGLT2i therapy in hemoglobin, hematocrit, albumin, AST, ALT, ALP, GGT, or body weight. Patients with FCFpEF had significantly lower overall hemoglobin (−1.0 mmol/L, *p* = 0.025), hematocrit (−0.04 L/L, *p* = 0.025), and ALT (−7.4 U/L, *p* = 0.022) compared to FCFrEF patients. The two patients with PLE had a significantly lower overall serum albumin than patients without PLE (−9.6 g/L, *p* = 0.009). FCF phenotype did not significantly influence the other variables.

### Clinical outcomes

**Figure 3:**
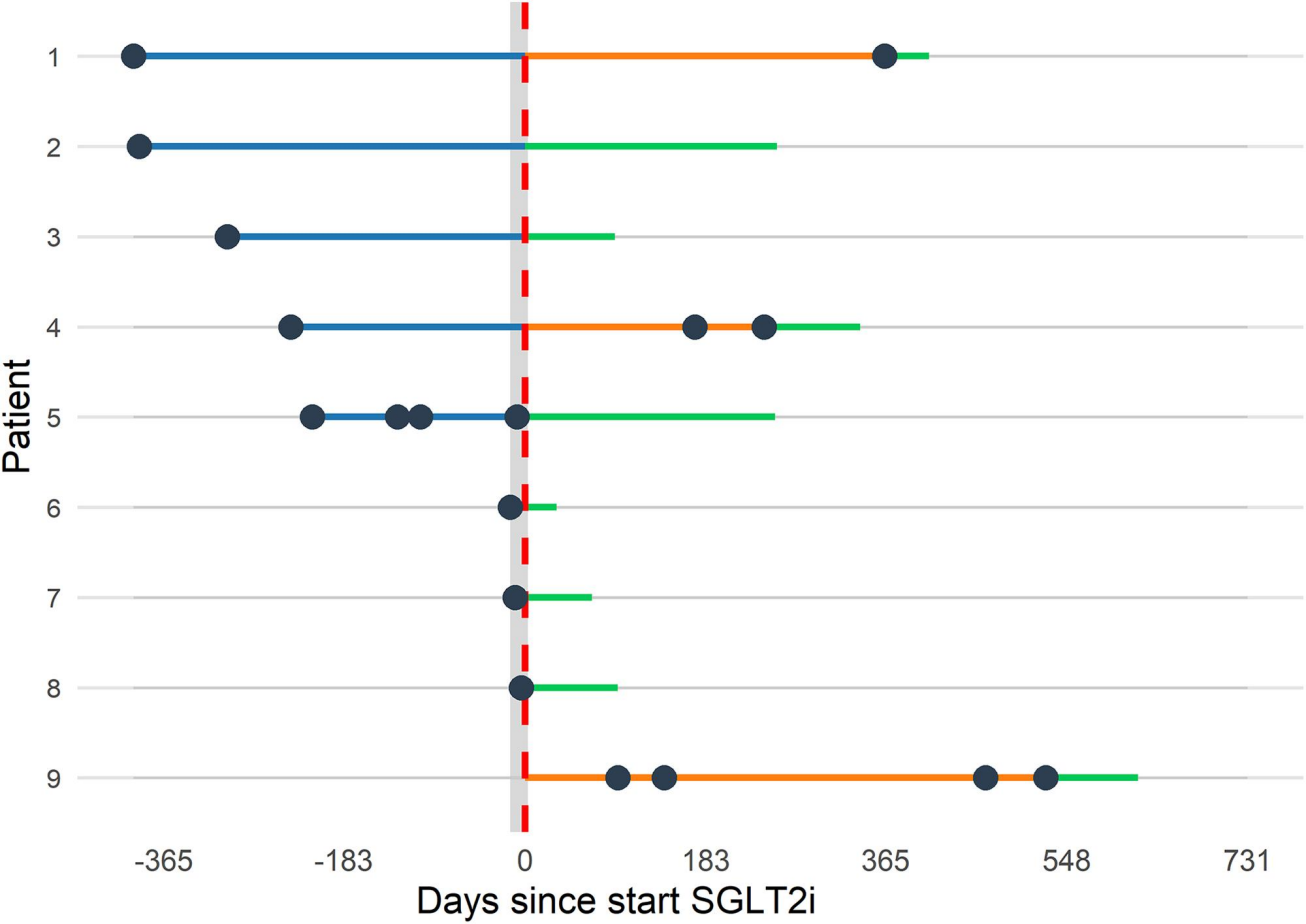
Timeline of FCF-related hospitalizations. Timeline of the occurrence of FCF-related hospitalizations in the year before starting SGLT2i (blue, *n* = 11) and during follow-up (orange, *n* = 7). The green lines indicate the time until the most recent follow-up without hospitalization. The dashed red line indicates when SGLT2i was started. Dots that fall within the light gray band indicate FCF-related hospitalizations during which SGLT2i was started (which was the case in patients 5, 6, 7, and 8). FCF, Fontan circulatory failure; SGLT2i, sodium-glucose cotransporter 2 inhibitors.

A total of 18 FCF-related hospitalizations, with a median length of stay of 5.5 [2.5–12.3] days, were documented in 9 patients; 11 in the year before starting SGLT2i, and 7 during follow-up. The timeline for the FCF-related hospitalizations is presented in Figure 3. Additionally, 2 patients underwent atrial ablation procedures for arrhythmias during follow-up, and 1 underwent lymphatic embolization. One patient with Loeys-Dietz syndrome died after emergency thoracic endovascular aneurysm repair for a type B aortic dissection. No other patients died during follow-up and none were lost to follow-up.

### Concomitant FCF pharmacotherapy changes

**Figure 4:**
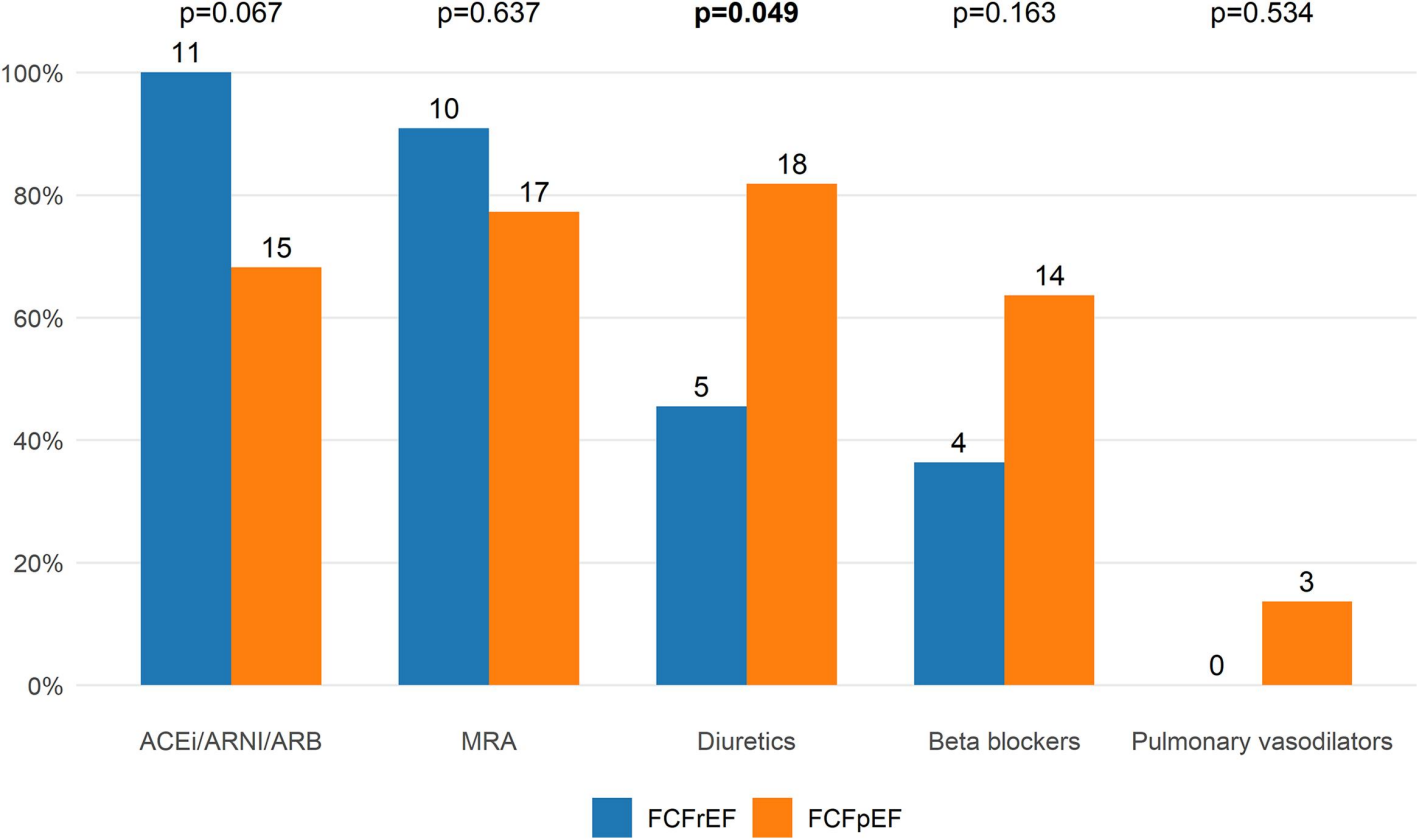
FCF pharmacotherapy at baseline. Proportion of patients using different FCF pharmacotherapy classes at baseline, separated for FCF type (FCFrEF *n* = 11, FCFpEF *n* = 22). The total number of patients using each pharmacotherapy class is shown at the top of each bar. FCFpEF patients more frequently used diuretics at baseline than FCFrEF patients (81.8% vs. 45.5%, *p* = 0.049). ACEi, angiotensin-converting enzyme inhibitor; ARB, angiotensin receptor blockers; ARNI, angiotensin receptor-neprilysin inhibitor; FCF, Fontan circulatory failure; FCFpEF, Fontan circulatory failure with preserved ejection fraction; FCFrEF, Fontan circulatory failure with reduced ejection fraction; MRA, mineralocorticoid receptor antagonist.

All but one patient (97%) used at least one of the “conventional” guideline-directed medical heart failure therapy classes at baseline, 17 patients (51.5%) used two, and 11 patients (33.3%) three medication classes when SGLT2i was started. The proportion of patients using each medication class at baseline, stratified for FCF phenotype, is presented in Figure 4. FCFpEF patients more frequently used diuretics than FCFrEF patients at baseline (81.8% vs. 45.5%, *p* = 0.049), while other medication classes did not differ significantly. From baseline to most recent follow-up, there were no significant changes in the use of any of the FCF pharmacotherapy classes (Supplementary Table 4).

## Discussion

This real-world multicenter study evaluated SGLT2i in the adult Fontan population. The most important findings are that SGLT2i were safe and well-tolerated in patients with FCF, and SGLT2i treatment was associated with a significant decrease in NT-proBNP, in both FCFrEF and FCFpEF patients.

### Safety and tolerability of SGLT2i in Fontan patients

In the current study, 5 patients (15.2%) experienced side effects, of whom 3 (9.1%) permanently discontinued SGLT2i therapy. These findings are in line with the previous report of 174 ACHD patients using SGLT2i (18). However, when directly comparing the results, we should acknowledge the influence of the small sample size on the reported percentages. Importantly, there were no new or serious side effects, and it is reassuring that no significant deterioration of blood pressure or renal function was observed. Therefore, these findings suggest that SGLT2i have a comparable safety profile and are tolerated well in the FCF population.

### NT-proBNP in the Fontan population

Several studies have reported a significant reduction in NT-proBNP after starting SGLT2i in ACHD patients with a biventricular circulation (20–23). To our knowledge, this is the first study to report a significant reduction in NT-proBNP among FCF patients after SGLT2i initiation. Although natriuretic peptides have established prognostic value in ACHD, their role in the Fontan population is less well-established (5, 24). There is some evidence that NT-proBNP levels vary between the Fontan circulation types, with atriopulmonary and atrioventricular connection Fontans exhibiting higher NT-proBNP levels than patients with a total cavopulmonary correction, likely caused by the chronic exposure of atrial tissue to supranormal pressures (25). Despite the inclusion of all Fontan subtypes in our study, an overall reduction in NT-proBNP was observed. Elevated NT-proBNP levels have been associated with significant ventricular dilatation, reduced ejection fraction, and long-term clinical events in Fontan patients regardless of the type of Fontan repair (26, 27). Supporting this, the recently published *Fontan Adult Brompton (FAB)* clinical score study demonstrated significantly higher NT-proBNP levels among patients who died or underwent transplantation compared with survivors, reinforcing its potential as a surrogate marker of long-term prognosis (28).

### Differential response to SGLT2i between FCF phenotypes

Distinctly different underlying pathophysiological mechanisms of FCF have been identified, and significant systolic ventricular dysfunction appears to be the most prevalent underlying cause of FCF (29). We hypothesized that the response to SGLT2i might be different between FCFrEF and FCFpEF patients, in part based on a recent study evaluating the echocardiographic effects of SGLT2i in patients with single ventricle circulatory failure. In that study, fractional area change only improved significantly in patients with at least moderately reduced ventricular function at baseline, although improvements in free wall strain and isovolumic acceleration were seen across all ranges of systolic ventricular function (15). Konduri et al. also distinguished between FCFrEF and FCFpEF patients and reported sequential natriuretic peptides only for five FCFrEF patients after starting SGLT2i. They concluded that SGLT2i could be particularly effective in FCFrEF patients with elevated natriuretic peptides (13). The current study differs from these findings and demonstrates a significant reduction in NT-proBNP in both FCFrEF and FCFpEF patients. Although based on a limited sample size, the association with a reduction in NT-proBNP across FCF phenotypes is promising. Nonetheless, treatment response may be heterogeneous, reflecting different pathophysiological mechanisms of FCF. As the ACHIEVE-SGLT2i registry data did not allow for detailed hemodynamic phenotyping, future research should focus on elucidating the effects of SGLT2i in the different pathophysiological phenotypes of FCF, including patients with significant atrioventricular valve regurgitation, elevated pulmonary vascular resistance/pressures, and/or restrictive physiology (29).

FCF is a complex cardiovascular syndrome, characterized by disruption in the delicate balance between ventricular preload and systemic afterload (5). Due to this balance, the systemic effects of SGLT2i could potentially be detrimental in some FCF patients. Despite this, the off-target effects may explain both improvements in systolic ventricular function and the improvements in NT-proBNP reported here (15). The contributions of the pleiotropic mechanisms of action of SGLT2i in FCF remain unknown, and future studies should explore how these various mechanisms of action contribute to benefits in distinct FCF phenotypes.

### Overall differences between FCF phenotypes

While no significant differences in the response to SGLT2i were observed between FCFrEF and FCFpEF patients in this study, FCFpEF patients had lower overall serum hemoglobin and hematocrit. Although these values remained within the normal reference ranges, it is important to note that reference values are lacking for Fontan patients. In particular, hemoglobin values for (mildly) cyanotic patients are significantly higher than for non-cyanotic patients, and normal or slightly decreased hemoglobin values may be inadequate for Fontan patients (30, 31). In conventional heart failure, patients with preserved ejection fraction often have a lower hemoglobin and higher prevalence of anemia compared to patients with reduced ejection fraction, and anemia is an independent risk factor for mortality in heart failure patients (32, 33). The estimated hemoglobin of 9.1 mmol/L in FCFpEF compared to 10.1 mmol/L in FCFrEF patients at SGLT2i initiation may represent a similar, underrecognized difference, and systematic screening for anemia (and underlying iron deficiency) should be included as part of routine clinical care.

The prognostic significance of ALT in heart failure is less clear (34, 35). While there is no literature on the differences in hepatic serum biomarkers between FCFrEF and FCFpEF patients, over 80% of Fontan patients exhibit abnormal hepatic serum biomarkers over time, and patients frequently develop Fontan-associated liver disease (FALD) (36). Although ALT values stayed within the normal range (10–45 U/L) in this study, and more detailed hepatic function information was not available, the overall higher ALT observed in FCFrEF patients could be reflective of more pronounced hepatic congestion and hepatic hypoxemic injury due to significant ventricular dysfunction (37).

### Clinical outcomes

After starting SGLT2i, 7 FCF-related hospitalizations occurred in just 3 patients, 2 of whom also had an FCF-related hospitalization in the 12 months before initiating SGLT2i. “Frequent admitters” are a well-described phenomenon in conventional heart failure, with repeated hospitalizations being a marker of advanced disease and worse outcomes. This pattern appears to be present in the FCF cohort described here (38). We have to emphasize that the reduction in FCF-related hospitalizations observed from the year before to after starting SGLT2i was influenced by the limited follow-up duration and limited sample size, precluding further analysis. Hence, these data should only be regarded as hypothesis-generating.

### Limitations

This study has several limitations. First, this study is limited by the single-arm, retrospective, real-world setup, and the anatomical and surgical heterogeneity inherent to the Fontan population. Linear mixed models were used to account for within-patient correlations and unbalanced data, with data from the year before starting SGLT2i serving as the patients' control data. While randomized controlled trials remain the gold standard, real-world registries provide essential evidence that advances clinical care in the Fontan population. Second, the lack of (exercise) catheterization data in the registry prevented detailed stratification of the underlying hemodynamic mechanisms of FCF. The dichotomization of patients into FCFrEF and FCFpEF oversimplifies the complex phenotypes of FCF, and future studies should aim to evaluate the benefits of SGLT2i for different underlying causes. Third, although all patients had preserved renal function at baseline and there was no deterioration after SGLT2i, creatinine-based eGFR estimation is influenced by skeletal muscle and may overestimate actual renal function in the Fontan population, which is known to be subject to sarcopenia. Twenty-four-hour urine creatinine clearance would have provided a more accurate estimate of renal function. Cystatin C-based eGFR estimation also appears more accurate in Fontan patients and may be more sensitive in detecting renal dysfunction and SGLT2i-related changes (39). Unfortunately, neither were performed routinely. Fourth, functional performance was not evaluated in this study due to the limited availability of exercise testing data in the registry, while it is very relevant for a comprehensive evaluation of the potential efficacy of SGLT2i in the Fontan population. Conventional maximal exercise parameters, such as VO_2_ max, may not fully capture “Fontan performance”, and therefore, submaximal exercise parameters may be of additional value in capturing clinically meaningful changes (40, 41).

## Conclusion

This real-world, multicenter study is the largest experience to date evaluating SGLT2i in adults with FCF. In this cohort of 33 patients, SGLT2i treatment was safe and well-tolerated, and associated with a significant reduction in NT-proBNP in both FCFrEF and FCFpEF phenotypes. Future research should confirm these observations in larger cohorts with longer follow-up, and focus on identifying patients who are most likely to benefit from SGLT2i treatment. Preferably, this should be done through prospective studies incorporating detailed hemodynamic phenotyping, to evaluate treatment response between the different underlying causes of FCF.

## Data Availability

The raw data supporting the conclusions of this article will be made available by the authors, upon reasonable request.
