# Supplementary material for: Real-world experience with sodium-glucose cotransporter 2 inhibitors in adults with Fontan circulatory failure
Source: Front Cardiovasc Med. 2026 May 8;13:1771868. doi: 10.3389/fcvm.2026.1771868 (PMC13194575; doi:10.3389/fcvm.2026.1771868)
Supplement: Supplementary file 1 [file Datasheet1.docx]

**SUPPLEMENTAL APPENDIX**

**Real-World Experience with Sodium-Glucose Cotransporter 2 Inhibitors in Adults with Fontan Circulatory Failure**

**Contents:**

|  | **Page** |
| --- | --- |
| **SUPPLEMENTAL TABLES** |  |
| **Supplemental Table 1.** Side effects of SGLT2i therapy | 2 |
| **Supplemental Table 2.** Mixed model outputs | 3 |
| **Supplemental Table 3.** Mixed model characteristics | 5 |
| **Supplemental Table 4.** Concomitant pharmacotherapy changes | 6 |
| **SUPPLEMENTAL FIGURES** |  |
| **Supplemental Figure 1.** Participating centers | 7 |

**SUPPLEMENTAL TABLES**

**Supplemental Table 1.** Side effects of SGLT2i therapy

|  | **n=33** |
| --- | --- |
| Number of patients who reported side effects | 5 (15.2) |
| *Documented side effects* |  |
| Fatigue | 2 (6.1) |
| Acute kidney injury | 1 (3) |
| Abdominal discomfort | 1 (3) |
| Dysuria | 1 (3) |
| Nausea | 1 (3) |
| Symptomatic (orthostatic) hypotension | 1 (3) |
| Urinary tract infection (uncomplicated) | 1 (3) |
| Values are presented as n (%). Data were available for all patients. *SGLT2i, sodium-glucose cotransporter 2 inhibitors.* | |

**Supplemental Table 2.** Mixed model outputs

|  | **Estimate** | **Standard Error** | **P-value** |
| --- | --- | --- | --- |
| **Safety** |  |  |  |
| *Creatinine* |  |  |  |
| Intercept | 73.272 | 2.556 | 0.000 |
| Time before start | 0.014 | 0.008 | 0.082 |
| Time after start | 0.007 | 0.015 | 0.648 |
| *Systolic blood pressure* |  |  |  |
| Intercept | 110.136 | 2.109 | 0.000 |
| Time before start | -0.021 | 0.013 | 0.101 |
| Time after start | 0.015 | 0.019 | 0.443 |
| *Diastolic blood pressure* |  |  |  |
| Intercept | 66.783 | 1.573 | 0.000 |
| Time before start | -0.001 | 0.009 | 0.894 |
| Time after start | -0.006 | 0.014 | 0.680 |
| **Efficacy** |  |  |  |
| *NT-proBNP*^*^ |  |  |  |
| Intercept | 2.436 | 0.091 | 0.000 |
| Time before start | <0.001 | <0.001 | **0.022** |
| Time after start | -0.001 | <0.001 | **0.010** |
| *Hemoglobin* |  |  |  |
| Intercept | 10.115 | 0.358 | 0.000 |
| Time before start | 0.001 | 0.001 | 0.224 |
| Time after start | <0.001 | 0.001 | 0.793 |
| FCF type (0=FCFrEF, 1=FCFpEF) | -1.022 | 0.432 | **0.025** |
| *Hematocrit* |  |  |  |
| Intercept | 0.483 | 0.014 | 0.000 |
| Time before start | <0.001 | <0.001 | 0.106 |
| Time after start | <0.001 | <0.001 | 0.656 |
| FCF type (0=FCFrEF, 1=FCFpEF) | -0.040 | 0.017 | **0.025** |
| *Albumin* |  |  |  |
| Intercept | 46.447 | 1.050 | 0.000 |
| Time before start | 0.006 | 0.004 | 0.097 |
| Time after start | -0.007 | 0.006 | 0.318 |
| PLE (0=no PLE, 1=PLE) | -9.639 | 3.422 | **0.009** |
| *AST* |  |  |  |
| Intercept | 26.191 | 1.643 | 0.000 |
| Time before start | 0.005 | 0.005 | 0.322 |
| Time after start | -0.006 | 0.009 | 0.523 |
| *ALT* |  |  |  |
| Intercept | 28.083 | 2.606 | 0.000 |
| Time before start | -0.002 | 0.005 | 0.710 |
| Time after start | 0.012 | 0.009 | 0.184 |
| FCF type (0=FCFrEF, 1=FCFpEF) | -7.420 | 3.059 | **0.022** |
| *ALP*^*^ |  |  |  |
| Intercept | 1.955 | 0.030 | 0.000 |
| Time before start | <-0.001 | <0.001 | 0.921 |
| Time after start | <0.001 | <0.001 | 0.255 |
| *GGT*^*^ |  |  |  |
| Intercept | 1.816 | 0.051 | 0.000 |
| Time before start | <0.001 | <0.001 | 0.277 |
| Time after start | <0.001 | <0.001 | 0.426 |
| *Weight* |  |  |  |
| Intercept | 73.426 | 2.032 | 0.000 |
| Time before start | 0.005 | 0.005 | 0.379 |
| Time after start | <0.001 | 0.008 | 0.999 |
| Estimates are displayed as intercept values or temporal coefficients of the predicted changes per day. ^*^These models were fitted on a log10 transformed variable due to significant departure from normality of the normalized residuals for the normal scale variable. Model estimates and standard errors are in the log10 scale. *ALP, alkaline phosphatase; ALT, alanine transaminase; AST, aspartate transaminase; GGT, gamma-glutamyltransferase; NT-proBNP, N-terminal pro–B-type natriuretic peptide.* | | | |

**Supplemental Table 3.** Mixed model characteristics

|  | **Log transformation** | **Correlation structure** | **Variance structure** | **Covariate** |
| --- | --- | --- | --- | --- |
| **Safety** |  |  |  |  |
| Creatinine | No | Unspecified | VarIdent for intervention | NS |
| Systolic blood pressure | No | Unspecified | Unspecified | NS |
| Diastolic blood pressure | No | Unspecified | Unspecified | NS |
| **Efficacy** |  |  |  |  |
| NT-proBNP | Log10 | Unspecified | VarPower | NS |
| Hemoglobin | No | corExp | Unspecified | FCF phenotype as fixed effect |
| Hematocrit | No | corGaus | Unspecified | FCF phenotype as fixed effect |
| Albumin | No | corExp | Unspecified | PLE as fixed effect |
| AST | No | Unspecified | Unspecified | NS |
| ALT | No | corGaus | VarExp | FCF phenotype as fixed effect |
| ALP | Log10 | corCAR1 | VarPower | NS |
| GGT | Log10 | Unspecified | VarIdent for intervention | NS |
| Weight | No | corSpher | VarIdent for intervention | NS |
| All models were constructed as linear mixed models with random intercepts, time as a continuous linear variable ranging from -365 to + 365 days, and with a binary interaction term for the intervention (on SGLT2i yes or no). *ALP, alkaline phosphatase; ALT, alanine transaminase; AST, aspartate transaminase; FCF, Fontan circulatory failure; GGT, gamma-glutamyltransferase; NS, not significant; NT-proBNP, N-terminal pro–B-type natriuretic peptide; PLE, protein-losing enteropathy; SGLT2i, sodium-glucose cotransporter 2 inhibitors.* | | | | |

**Supplemental Table 4.** Concomitant pharmacotherapy changes

|  | **Baseline** | **Most recent follow-up**^*^ | **Most recent follow-up  p-value** |
| --- | --- | --- | --- |
| MRA | 27 (81.8) | 27 (81.8) | 1.000 |
| ACEi / ARNI / ARB | 26 (78.8) | 24 (72.7) | 0.500 |
| Diuretic | 23 (69.7) | 25 (75.8) | 0.500 |
| Beta blocker | 18 (54.5) | 20 (60.6) | 0.625 |
| Pulmonary vasodilator | 3 (9.1) | 4 (12.1) | 1.000 |
| Paired data of all patients were available. McNemar’s tests were used to compare baseline to most recent follow-up data. ^*^The median follow-up duration was 8.0 [3.2 – 12.2] months. *ACEi, angiotensin-converting enzyme inhibitor; ARB, angiotensin receptor blocker; ARNI, angiotensin receptor-neprilysin inhibitor; MRA, mineralocorticoid receptor antagonist.* | | | |

**SUPPLEMENTARY FIGURES**

**Supplemental Figure 1.** Participating centers

**
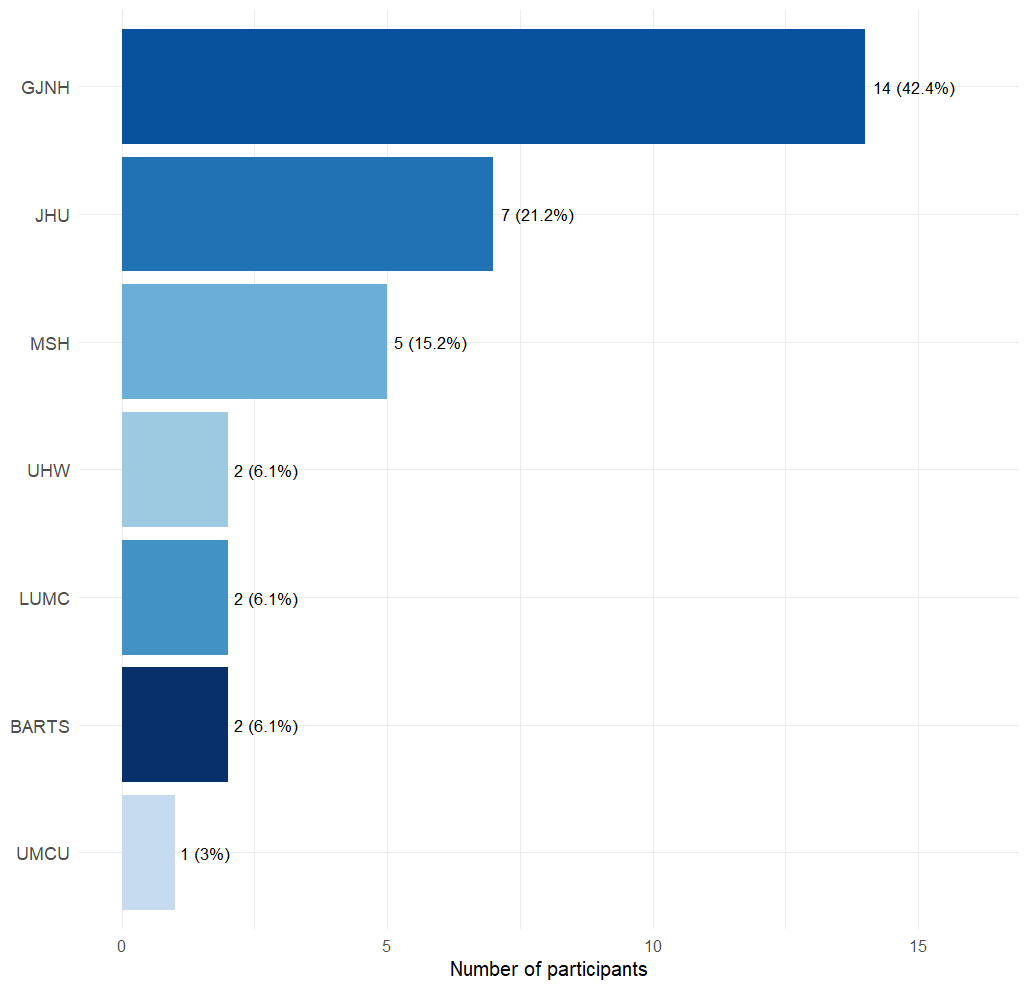
**

**Supplementary Figure 1 Legend.** Overview of the inclusions per participating center. *BARTS, Barts Heart Centre; GJNH, Golden Jubilee University National Hospital; JHU, Johns Hopkins University; LUMC, Leiden University Medical Center; MSH, Mount Sinai Fuster Heart Hospital; UHW, University Hospital of Wales; UMCU, University Medical Center Utrecht.*
